# A Forgotten Migrated Intrauterine Contraceptive Device Is Not Always Innocent: A Case Report

**DOI:** 10.1155/2010/740642

**Published:** 2010-08-25

**Authors:** Ranjeet Brar, Sudeendra Doddi, Anand Ramasamy, Prakash Sinha

**Affiliations:** Princess Royal University Hospital, Farnborough Common, Orpington, London BR6 8ND, UK

## Abstract

The incidence of transuterine perforation and migration of intrauterine contraceptive devices (IUCDs) into the abdominal cavity has been estimated at less than 0.1%. It has been suggested that intraperitoneal IUCD have low morbidity and may be left in situ. We report the first case of closed loop small bowel obstruction due to migration of a “*Saf-T-Coil*” IUCD into the abdominal cavity, where it became embedded in the omentum and ultimately, 31 years after deployment, coiled both arms around a loop of ileum. This late complication underlines the dangers of intra-abdominal foreign bodies, even when chemically and biologically inert.

## 1. Case Report

A 64-year-old post-menopausal lady presented with a one day history of vomiting and severe right iliac fossa pain. This had been preceded by 2 months history colicky lower abdominal pain for which she had not sought medical advice. She continued to vomit in the ED and on admission (totalling 5 episodes in 24 hours).

Her past surgical history consisted of open appendicectomy at 21 years of age, termination of pregnancy at 31 years, and a laparoscopic tubal ligation at the age of 38. (She underwent menopause at the age of 54.)

A “*Saf-T-Coil*” [Julius Schmid Laboratories, Little Falls, New Jersey, USA. Manufactured 1967–1982] IUCD was placed when she was 30 years old [1]. She became pregnant within the year, however, and was counselled that there was increased risk of miscarriage and perinatal mortality in the presence of the IUCD. For this reason she opted to have her pregnancy terminated. The cervical thread was not apparent at the os cervix, and the IUCD was not encountered during the termination procedure. Postoperatively, she was informed that the device had “fallen out”, and was discharged without further investigation. She had no other past medical history of note.

On Examination, she was apyrexial and haemodynamically stable with a pulse of 78/min and a BP of 110/74 mmHg. Other than a respiratory rate of 24, her cardiorespiratory examination was unremarkable. Her abdomen was obviously distended, and while it was soft, there was marked right iliac fossa tenderness. WBC was 16.6 × 10^9^/L, and CRP was 4. Liver function tests, urea, and electrolytes were within normal limits.

**Figure 1:**
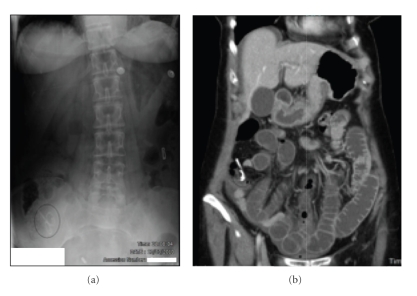
(a) Abdominal X-ray. No free intra-abdominal air. Note IUCD in RIF. (b) CT scan of the abdomen.

**Figure 2:**
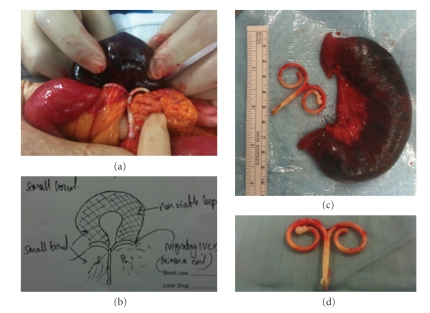
(a) Intra-operative photograph showing migratory “*Saf-T-Coil*” IUCD, with each arm wrapped around a loop of mid ileum. (b) Operative note depicting configuration of “*Saf-T-Coil*” IUCD causing closed loop bowel obstruction. (c) Resected specimen-ischaemic loop of small bowel. (d) Obstructing “*Saf-T-Coil*” IUCD removed from abdomen.

Her abdominal radiograph (AXR) taken on presentation is shown in Figure 1(a). The patient's obstructive symptoms were correlated with radiological findings and an apparently ectopic IUCD was noted. In light of the clinical and radiological findings, computed tomography was performed (see Figure 1(b)). This demonstrated obstruction of the small bowel in association with the migratory IUCD, and the patient underwent laparotomy to relieve the obstruction. Intraoperative findings are depicted in Figure 2.

The migratory “*Saf-T-Coil*” IUCD, fitted some 31 years earlier, had become embedded in the omentum, and each of the two “arms” of the coil had encircled the lumen of a segment of mid ileum, giving rise to a closed loop obstruction. A segmental resection was performed with stapled side-to-side (functional end-to-end) anastamosis. The uterus was small and retroverted, without obvious scarring. Postoperative recovery was unremarkable and pathology revealed a 14 cm segment of infarcted bowel.

## 2. Discussion

Intrauterine contraceptive devices (IUCDs) are the worlds most widely used method of reversible birth control. Their modern use dates from 1909, but a high rate of intrauterine infection led to their withdrawal until redesigned from inert materials and reintroduced in the late 1950s [2]. Complications include pain, bleeding, a failure rate in the order of 4% (conception despite correct deployment or accidental expulsion from the uterus—usually along the trajectory of insertion, via the cervix and vagina), an increased rate of pelvic inflammatory disease (PID) and toxic shock syndrome [3]. Colonic obstruction has also been described in the context of severe pelvic inflammatory disease (PID) with prolonged IUCD use [4, 5]. 

Incidence of uterine perforation is estimated to be less than 0.1%, and is a consequence of uterine injury at the time of insertion in most cases [6, 7]. Early puerperal insertion (within 12 weeks of delivery) and pregnancy in the presence of an IUCD have been advanced as putative risk factors for uterine perforation, in addition to insertion technique [8]. 

The fate of IUCD once they have entered the abdominal cavity varies. The majority perforate the uterus completely and most remain in the pelvic cavity; a significant minority become embedded in the omentum [7]. Early closed loop devices (such as the Birnberg bow) were associated with small bowel strangulation, and were discontinued for this reason. Copper coils induce marked peritoneal reaction, causing adhesion formation and bowel injury [9]. Lippes Loops and Saf-T-Coils are inert, “nonmedicated” devices that cause little biological reaction, and can be quiescent for long periods, as this case demonstrates. It has been suggested that modern intraperitoneal IUCD have low morbidity and may be left in situ [10]. 

While 85% of reported cases of uterine perforation have not caused major complications at the time of diagnosis, 15% have presented with serious complications of visceral perforation, with IUCD eroding partially or completely into the bladder, small bowel, appendix, colon, or rectum. Recto-uterine fistula and rectal stricture have also been reported [7, 9]. Ectopic IUCD in the presence of fever, abdominal pain, or diarrhoea should alert the clinician to the possibility of bowel perforation. Small bowel obstruction is an extremely rare presentation of open loop IUCD.

Visceral complications have been reported at a median time interval of 17 months (varying from four weeks to 13 years) [9]. This case demonstrates that small bowel obstruction can occur as late as 31 years following intra-abdominal translocation of an inert and ‘open loop’ *Saf-T-Coil* IUCD, due to direct strangulation of the bowel by the device.

In the case of partial uterine perforation or other visceral involvement, careful pre-operative CT imaging and planning is of great value. When IUCD ectopy is diagnosed in an as yet asymptomatic patient, one should be aware that late complications can occur in rare cases, and consider elective removal.
